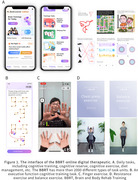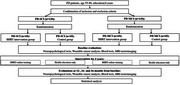# A Four‐Arm Randomized Controlled Trial on the Effects of Multi‐Dimensional Cognitive Intervention in Parkinson's Disease Patients with Mild Cognitive Impairment and Subjective Cognitive Decline (REMIND)

**DOI:** 10.1002/alz70860_098518

**Published:** 2025-12-23

**Authors:** Pei Huang, Chao Gao, Yuyan Tan, Shengdi Chen, Yun Ling, Zhonglue Chen, Zhixing Zhou, Huanhuan Xia

**Affiliations:** ^1^ Ruijin Hospital, Shanghai Jiao Tong University School of Medicine, Shanghai, China; ^2^ GYENNO SCIENCE Co., Ltd, Shanghai, China; ^3^ Shanghai Bestcovered Limited, Shanghai, China

## Abstract

**Background:**

Although there is currently no effective pharmacological treatment to delay the progression of mild cognitive impairment (MCI) to dementia, accumulating evidence supports that multi‐domain interventions, including cognitive training, exercise, and dietary modifications, offer a promising approach. Emerging digital therapies, incorporating diverse audio and visual stimuli, can be integrated into these multi‐domain interventions to further enhance cognitive benefits. This study aims to investigate the efficacy of online Brain and Body Rehab Training (BBRT) (Figure 1), a digital cognitive intervention, in Parkinson's disease patients with mild cognitive impairment (PD‐MCI) and subjective cognitive impairment (PD‐SCI).

**Method:**

This study is a 3‐year, four‐arm, randomized controlled trial designed to recruit 130 PD‐MCI patients and 130 PD‐SCI patients, aged 55‐80, with at least 6 years of education, and no other neurological disorders or serious systemic diseases. Participants will be randomly assigned to either the BBRT intervention group (*n* =65) or the control group (*n* = 65). The control group will receive regular health education as the BBRT intervention group, while the BBRT intervention group will undergo at least five online BBRT training sessions per week, with each session lasting a minimum of 15 minutes, conducted remotely. Both groups will receive their respective interventions for 36 months, with annual follow‐ups to assess the efficacy of BBRT (Figure 2).

**Result:**

The primary outcome of this study will be the total score on the Montreal Cognitive Assessment (MOCA). Secondary outcomes include: (1) Neuropsychological scales: MMSE, PD‐CRS, HAMA, HAMD, PDQ‐39, and MDS‐UPDRS scales. (2) Wearable sensor‐based digital biomarkers: Gait, speech, and handwriting analysis. (3) Blood biomarkers: Aβ42, T‐tau, *p*‐tau, and neuroinflammatory markers. (4) Neuroimaging outcomes: Structural and functional MRI changes. All participants will be assessed at baseline, and at 12, 24, and 36 months for these outcomes. The research protocol has been approved by the Ethics Committee of Ruijin Hospital, affiliated with Shanghai Jiao Tong University School of Medicine (KY2024‐137).

**Conclusion:**

This study will systematically assess the clinical efficacy of BBRT‐online intervention in Chinese PD‐MCI and PD‐SCI patients and provide valuable insights into the mechanisms of non‐pharmacological interventions for enhancing cognitive function.